# Genome hyperevolution and the success of a parasite

**DOI:** 10.1111/j.1749-6632.2012.06654.x

**Published:** 2012-09-06

**Authors:** J David Barry, James P J Hall, Lindsey Plenderleith

**Affiliations:** Wellcome Trust Centre for Molecular Parasitology, Institute of Infection, Immunity and Inflammation, College of Medical, Veterinary, & Life Sciences, University of GlasgowGlasgow, United Kingdom

**Keywords:** genome hyperevolution, antigenic variation, parasite, trypanosome, subtelomere

## Abstract

The strategy of antigenic variation is to present a constantly changing population phenotype that enhances parasite transmission, through evasion of immunity arising within, or existing between, host animals. Trypanosome antigenic variation occurs through spontaneous switching among members of a silent archive of many hundreds of variant surface glycoprotein (*VSG*) antigen genes. As with such contingency systems in other pathogens, switching appears to be triggered through inherently unstable DNA sequences. The archive occupies subtelomeres, a genome partition that promotes hypermutagenesis and, through telomere position effects, singular expression of *VSG*. Trypanosome antigenic variation is augmented greatly by the formation of mosaic genes from segments of pseudo-*VSG*, an example of implicit genetic information. Hypermutation occurs apparently evenly across the whole archive, without direct selection on individual *VSG*, demonstrating second-order selection of the underlying mechanisms. Coordination of antigenic variation, and thereby transmission, occurs through networking of trypanosome traits expressed at different scales from molecules to host populations.

## Introduction

Parasites and their hosts notoriously engage in an arms race, evolving measures and countermeasures against each other in a battle for supremacy. So strong are the selective forces, and consequently the rate and extent of evolution, that we can see a variety of extreme adaptations that are informative of mutational mechanisms and of general eukaryotic biology. Highly prominent in the arms race are phenotypes associated with evasion of mammalian immunity. Escape by a parasite might seem relatively simple—hide, or perhaps block, a key step in immunity, or maybe adopt camouflage—but closer inspection often reveals a highly complex biological system. The system often includes adaptations at various scales, ranging from the genome, through cellular structures and machinery, to population behavior within and between infections. Furthermore, the functional outcomes of such adaptations generally are coordinated, forming a network of interacting processes that underpin a long-term strategy for enhanced transmission of the parasite and hence its persistence in an ecosystem. Nowhere is this complex type of evasion system more apparent than in the antigenic variation of African trypanosomes.

## Antigenic variation: tactics for population survival

Antigenic variation is fundamental to the success of many pathogens. As infection proceeds, the resident pathogen population is decimated by populations of highly specific antibodies directed typically against its major surface antigen. Some individuals, however, have already switched to antigenically different versions of that molecule and survive and proliferate, a process that repeats many times, producing alternating waves of antigens and antibodies.^1^ This process reflects the classical arms race in which pathogen survival mechanisms and host immune mechanisms coevolve. With the capacity to make ∼10^10^ distinct antibody idiotypes, mammals seem destined to win this race. This enormous potential for immunological variation cannot be realized simply through direct encoding of antibodies in our genome, and requires a set of mechanisms that differentially shuffle and combine encoded components of antibodies.^2^ How can a parasite, with a much smaller and less complex genome, compete?

In considering this question, we must broaden our vision. We have to adopt a systems biology approach, in which we link different functional scales of the parasite. Within infections, antigenic variation operates in concert with other intrinsic parasite parameters including growth and differentiation dynamics, to name just two.^3^ Beyond individual infections, parasites have to be transmitted to new hosts. For a parasite that is abundant in the field, there is not an endless supply of new hosts, so a major challenge is likely to be successfully reinfecting hosts that already have antibodies corresponding to previous infections, either in circulation or stored as memory. Within an evolutionary framework, interactions at all scales, from molecules to host populations, amalgamate into a complex phenotype under complex selection. Features of antigenic variation, from molecules to parasite populations, will reflect the pressures of those selective forces.^3^

Trypanosomes live extracellularly in the blood, exposed to several immune mechanisms.^4^,^5^ Their variant surface glycoprotein (VSG) forms a dense coat that thwarts innate immunity and prevents antibodies accessing invariant cell surface molecules and eliminating the infection.^6^ A key event in the evolution of VSGs appears to have been dispensing with specific biochemical function, allowing their sequence to vary enormously, with merely some general structural and processing constraints. Spontaneously, at a rate of ∼10^−3^ switch/cell/generation, trypanosomes switch from one VSG to another, using mechanisms described below. The spontaneity and high rate of switching mean constant presence of variants before the onset of specific antibodies. For example, the first wave of parasites in a cow can peak with a total in excess of 10^11^ trypanosomes, and therefore >10^8^ will have switched to a different variant, while a mouse can support >10^8^ parasites with >10^5^ emerging variants.^7^ Many microbes facing strong challenges in hosts display such preemptive phenotypic variation, arising specifically from highly mutable contingency loci.^8^

In the trypanosome system, VSG switching is coordinated with growth and density-dependent differentiation of the parasite from proliferative to the nonproliferative transmission phase, which will infect tsetse flies on uptake in a bloodmeal.^9^ There is a fairly constant supply of the transmission stage, the level of which is critically affected by the number of easily activated VSG variants. The more VSG variants that are present at a time, the less chance each variant subpopulation has of reaching the threshold to induce an antibody response, meaning that, when the number of variants is high, overall population numbers are predicted to be controlled by differentiation.^3^,^10^ Such a swing toward differentiation-based control over immunity-based control results in higher levels of the transmission stage, but complete reliance on this mechanism would lead to prolonged high parasitemia and, therefore, earlier host death. In parallel, variants appear in ordered progression,^1^ which favors efficient use of the archive and promotes chronicity of infection. Ordered expression could be achieved by variants clustering into “blocks” of distinct activation probability, with each block corresponding to a growth peak in the host.^3^ As infections can run for years, many VSGs are probably required over the course of a single infection. Furthermore, it has been proposed that expressing substantially different subsets of VSGs would enable successful reinfection of previously exposed, partially immune hosts.^11^ Reinfection would be facilitated also by the known diversity in the “metacyclic” VSGs expressed by the initial population injected by the tsetse fly vector.^12^ On top of this reinfection requirement, the wide host range of trypanosomes introduces another dimension of uncertainty with which parasites must cope. Success in the wild, therefore, requires the various components of the network to adjust, enabling success in numerous host types.

## Multiple mechanisms contribute to the generation of VSG diversity

The trypanosome genome appears to have evolved to provide such flexibility. To do so has required unusual adaptations, which operate at different levels.

### Adaptations in genome structure

#### Expanded coding capacity and complexity

The enormous scale of antigenic variation is templated by an archive of nearly 2000 silent *VSG* genes and pseudogenes—nearly one-third of the core trypanosome genome.^13^,^14^ The archive is very diverse, particularly in the sequences encoding the N-terminal domain of the VSG, which carries the key epitopes seen by the immune system. Typically, amino acid identity in that domain between VSGs is less than 20%, a particularly broad range that arises presumably from the lack of specific biochemical function and because many common amino acids specify alpha-helix, which comprises most of the domain.^6^

#### Partitioning of the genome: the importance of subtelomeres

As with many eukaryotes, the trypanosome genome is effectively partitioned into the core, containing genes under purifying selection and where most mutation is repaired, and subtelomeres, where multigene families associated with organismal phenotypic variation diversify rapidly.^15^,^16^ The entire repertoire of *VSG*, including the archive and expressed genes, is located in subtelomeres.^14^ Trypanosome subtelomeres are relatively enormous, in one case being several times longer than the associated chromosome core.^17^

Functionally, subtelomeres contribute two special features relevant to *VSG* and antigenic variation. The first of these features is ectopic recombination. Unlike chromosome cores, subtelomeres recombine ectopically, permitting sequence exchange or modification between different chromosomes, introducing diversity.^18^ A number of recombination pathways can be involved.^19^ Other, more general, mutational mechanisms contribute to *VSG* diversification, but ectopic recombination plays a particularly significant role and is likely to be specific to subtelomeres.

The second feature is the telomere position effect, in which gene promoters close to elomeres are subject to reversible repression mediated by silencing proteins that bind, directly or indirectly, to the telomere tract at the chromosome end. In the trypanosome, this effect appears to have evolved further into a sophisticated regulatory system. The only loci from which *VSG* can be transcribed are expression sites (ES), of which there are several in the genome.^20^ All are telomere-proximal (i.e., the last genes before the telomere). Each ES comprises a promoter, several non-*VSG* genes, a set of imperfect repeats (“70-bp repeats”), and the *VSG* adjacent to the telomere. Antigen switching involves mostly replacement, by gene conversion, of the expressed *VSG* copy (or a part thereof), by all (or part) of another *VSG.*^1^,^21^ Only one ES is active at a time, which requires occupancy of a specific nuclear niche, the ES Body (see below), and inactivity of the other ES involves a telomere position effect.^22^ It is likely that telomere proximity also functions in antigen switching, due to the capacity of telomeres to interact with one another, promoting recombination.^18^

#### Supernumerary chromosomes

The trypanosome has evolved a set of ∼100 nuclear minichromosomes, which carry, with little exception, only telomere-proximal *VSG* genes.^23^ The two consequences are increase in archive size and availability of telomere-proximal *VSG*, possibly increasing the pool of telomere-proximal *VSG* that can interact readily with the expression site (switching) or with each other (recombination-mediated diversification).

#### Adaptations in nuclear structure and function

Trypanosome telomeric regions, including *VSG* genes, are located in heterochromatin at the nuclear periphery.^24^ *VSG* transcription is mediated by RNA polymerase I (RNAPI), which normally transcribes only ribosomal RNA genes located in the nucleolus. The active *VSG* occupies the ES Body, an RNAPI-containing extranucleolar niche located within the nuclear interior rather than peripherally.^25^ The minichromosomes behave differently from the core chromosomes, lying in the nuclear periphery, being duplicated earlier, and segregating via core microtubules rather than the spindle; the unusual behavior might be necessitated, at least partially, by overloading of the conventional machinery.^25^

#### Recombination prone sequences

Recombination and repair pathways serve fundamental functions in any organism, but they can be exploited to achieve specific phenotypes, such as in antigenic variation. One common mechanism is that an elevated rate of recombination causes the expressed gene to become replaced or altered. The elevated rate is specific to the contingency loci and is facilitated by the presence of recombination-prone sequences.^26^,^27^ In the *VSG* system, the unstable, 70-bp repeats lying upstream of the expressed *VSG* are thought to precipitate a high level of recombination (see below). It is also apparent that the subtelomere compartment in the trypanosome genome differs from the chromosome cores in its interaction with recombination/repair mechanisms, becoming disproportionately disrupted in null mutants of key players MRE11 and BRCA2.^28^,^29^ As to which recombination/repair pathways mediate *VSG* interactions, some interesting novelties exist in the trypanosome general pathways, but a formal link has yet to be made.^30^

#### Implicit information: pseudogenes and the combinatorial creation of novel intact genes

Some two-thirds of the array *VSG*s are pseudogenes, with premature stop codons, frameshifts, deletions, or lack of an appropriate start codon.^14^ At first sight, this situation appears to be a colossal waste of resources, but in fact it is probably the opposite. By what is likely to be nonreciprocal recombination (gene conversion) of wild-type fragments of pseudogenes into the expressed *VSG*, antigenically distinct genes can be generated.^31^ The pseudogenes therefore encode implicit information.^32^ Combinatorial use of silent information has potential for greatly expanding the extent of resulting, explicit information, as is demonstrated in the case of the bacterium *Anaplasma*, which can create apparently hundreds of antigenically distinct MSP2 variants from an archive of merely five unique pseudogenes, and no intact genes.^33^ The trypanosome pseudogene pool is more than two orders of magnitude greater in size. That short conversion fragments can introduce novel function is apparent also from the human HLA and KIR cell surface proteins of the human immune system.^34^–^36^

### How the antigenic variation phenotype is served by genome adaptations (Table 1)

**Table 1:** Genome features contributing to the antigenic variation phenotype*a*

Phenotype	Genome feature
Many VSGs	Large subtelomeres populated by *VSG* arrays
Singular VSG expression	ES Body: specialized nuclear niche;
	silencing through telomere position effects
Switching VSG	Telomere proximity of expression site enables interaction with *VSG* in other (sub)telomeres
Ordered expression—general	Locus types within subtelomeres have different activation probabilities
Ordered expression—specific	Flanks of each *VSG*?
	For mosaic *VSG* expression, coding sequence identity between donor and expressed genes
Antigenically diverse growth peaks	Blocks of distinct *VSG* with similar activation probability
Coordinated growth, differentiation, and antigenic variation	Number of *VSG* constituting a block. Higher number tends toward differentiation-based control; lower number favors antibody-mediated control
Prolonged infection	Mechanisms for ordered expression
	Mosaic *VSG* formation
Reinfection, superinfection	Diversity in *MVSG* set expressed in infective, metacyclic population?
	Mosaic *VSG* formation
	Contents and organization of blocks?
Host range	Large size of archive and its organization into blocks allow it to be used flexibly in different within-host environments, providing optimum transmission phenotype in each host species?
*VSG* hyperevolution	Enhanced mutation/reduced mutation repair in subtelomeres

a Speculation is denoted by question marks.

#### Singular VSG expression

The ES Body provides expression to one ES at a time, and a telomere position effect, possibly along with other epigenetic effects, provides silencing of the others, ensuring controlled expression of the archive.

#### Switching arises possibly from inherent tendency for double-strand breaks

The 70-bp repeats upstream of the transcribed *VSG* each contain a tract of the physically unstable motif (TAA)_n_.^37^ Instability of this type theoretically can cause stalling of DNA replication, leading in effect to production of a double-strand break (DSB) in the newly synthesized duplex and, consequently, induction of repair.^38^ A common route taken is creation of a gap and filling by copying from a homologous sequence. In this case, homology at another run of 70-bp repeats, upstream of a different *VSG,* would initiate the copying of that gene into the expression site. There is indeed some evidence that artificial creation of a DSB adjacent to the 70-bp repeats prompts switching by duplication, and that DSB occur naturally in the repeats in the expression site.^39^ As with other contingency loci systems, where runs of short repeats are thought to cause gene inactivation or reactivation through indels arising by DNA polymerase slippage on the repeat template,^8^ this mechanism is simple—an accident waiting to happen, at reasonably high frequency, due to inherent instability in the DNA sequence.

#### Ordered VSG expression

Antigenic variation runs in a pattern often termed *semipredictable*, with variants tending to appear in the same general order in distinct infections.^1^ Accomplishing order for such an enormous archive seems inordinately difficult, but it is facilitated in part by the genome environment and structure. Each gene appears to have an inherent activation probability. Those with the highest probability will activate continuously, but early onset of antibodies against their product will render all subsequent activations futile. The process will repeat as the activation hierarchy is unrolled. In general, telomere-proximal genes have the highest activation probability, followed by intact genes in the arrays, and then pseudogenes.^1^ Presumably, the BES telomere will interact more frequently with a donor at another telomere, via standard telomere associations, than with a donor in the subtelomere, which will require more routine homology searching. When mosaic gene formation comes into play, the interaction of donor with the BES is driven by degree of identity in the *VSG* coding sequence, making switching dependent on what went before.^14^

This gross level of order is refined, with blocks of variants appearing within each locus category. Whereas the degree of coding identity will specify finer order for mosaic gene formation, we do not know how refinement is achieved for the other genes. A clue, however, lies in the *Borrelia* antigenic variation system, where fairly exquisite ordering is achieved via two sequences, one in either flank of the gene.^40^ It is conceivable that the conserved sequences flanking *VSG* might have similar influence.

#### Diverse VSG expression

Each peak comprises a mixture of variants, the number of which is probably critical to the balance between antibody-mediated and density-dependent limitation of growth.^3^ In addition, diversity within each of these blocks decreases the extent to which antibody responses might eliminate the infection. Diversity probably occurs by all variants in a block having very similar activation probabilities.

#### Prolonged infection, reinfection, and superinfection

The combination of order and enormous potential for combinatorial creation of novel, mosaic expressed *VSG* can lead, at least in theory, to infections lasting months or even years. It has been hypothesized that the combinatorial mechanism also can allow reinfection of hosts already immune to that strain.^11^ Experimental analysis has shown that mosaic gene formation does enable reinfection of calves with the bacterium *Anaplasma.*^41^ Comparative genomics has shown that *VSG* archives are strain specific,^42^ a situation that enables strains to compete with each other in coinfections. In turn, competition will drive toward greater evolution of the archive.

#### Hyperevolution: selection of mechanisms that generate broad diversity

Second-order (indirect) selection^43^ underpins evolution of antigenic variation. Every *VSG* is dispensable, and most, being very rarely expressed, have little exposure to direct selection via their encoded protein. Yet, diversity appears to be spread evenly over the archive, for example with no evident outgrouping of the high-probability activators. Typically for multigene families in subtelomeres, the archive evolves rapidly. It seems that second-order selective processes operate, in which mutational mechanisms generate diversity across the whole *VSG* archive and across the whole coding sequence within each gene, independently of the immediate phenotype;^14^,^42^,^44^,^45^ it is these mechanisms that have been selected. Thus, an individual trypanosome with a novel trait that enhances population survival will be selected, but the dominant selective pressure acts at the level of the lineage, yielding mechanisms that directly confer benefit on the population rather than the individual. The view that evolution acts on only the immediate phenotype, rather than on long-term strategy is not compatible with what is observed for trypanosomes.

It is likely that the subtelomere provides an ideal environment for hypermutation, through naturally ectopic interactions and possibly through differential positioning within the nucleus. It is more likely that standard recombination-repair activities are used differentially on subtelomeres than that exclusive mutagenic mechanisms have evolved. *VSG* genes mutate in various ways. Ectopic gene conversion events duplicate *VSG* between subtelomeres, either as blocks of genes or individually.^14^ Recently, comparative genomics of sequential isolates of one trypanosome strain have revealed a range of mutations, including base substitutions, short indels, and conversions within the coding sequence (L.P., T. Otto, M. Berriman, and J.D.B., unpublished).

## Summary

To return to our question of how a parasite, with a smaller and less complex genome than that of its mammalian host, can compete in the face of antibody population diversity, the answer is flexibility. The trypanosome genome is adapted in somewhat extreme ways to serve the antigenic variation phenotype, with approximately 25% of the genome devoted to this phenomenon. The special features of subtelomeres, including hyperevolution and reversible gene-silencing mechanisms, have been exploited to great effect, resulting in an enormous archive of rapidly mutating silent *VSG* genes and telomere-associated mechanisms for their differential expression. Subtelomeres containing *VSG* arrays have expanded, and a large set of supernumerary chromosomes has emerged, apparently to provide even more telomeres. These structural adaptations have required changes in nuclear machinery. As with enhanced phenotypic variation systems in other pathogens, often referred to as *contingency gene systems*, the spontaneous phenotype switches that underpin antigenic variation appear to occur through inherent instability of a DNA sequence upstream of *VSG*. The silent *VSG* archive evolves at a rate several times faster than genes in chromosome cores, and it appears to do so without direct selection on expressed *VSG*. Instead, in a strong example of second-order selection, mechanisms have evolved that mutate all *VSG* apparently randomly. Much of the coding information is stored in the genome implicitly, in pseudogenes, requiring formation of mosaic genes for expression of novel antigens. The trypanosome *VSG* and mammalian antibody phenotypes both derive very extensive augmentation of information through the power of combinatorial reassembly of coding sequence. Besides these adaptations at the level of the genome, management of antigenic variation requires networking of *VSG* gene expression with other trypanosome phenotypes, including growth, differentiation, and, beyond the individual infection, unpredictable transmission into a very broad range of hosts, some of which will be partially immune.
